# Incidence and risk factors for Malaria, pneumonia and diarrhea in children under 5 in UNHCR refugee camps: A retrospective study

**DOI:** 10.1186/1752-1505-5-24

**Published:** 2011-10-26

**Authors:** Christine L Hershey, Shannon Doocy, Jamie Anderson, Christopher Haskew, Paul Spiegel, William J Moss

**Affiliations:** 1 Bloomberg School of Public Health, Johns Hopkins University, Baltimore, Maryland, USA; 2 United Nations High Commissioner for Refugees, Geneva, Switzerland

## Abstract

**Background:**

United Nations High Commissioner for Refugees (UNHCR) refugee camps are located predominantly in rural areas of Africa and Asia in protracted or post-emergency contexts. Recognizing the importance of malaria, pneumonia and diarrheal diseases as major causes of child morbidity and mortality in refugee camps, we analyzed data from the UNHCR Health Information System (HIS) to estimate incidence and risk factors for these diseases in refugee children younger than five years of age.

**Methods:**

Data from 90 UNHCR camps in 16 countries, including morbidity, mortality, health services and refugee health status, were obtained from the UNHCR HIS for the period January 2006 to February 2010. Monthly camp-level data were aggregated to yearly estimates for analysis and stratified by location in Africa (including Yemen) or Asia. Poisson regression models with random effects were constructed to identify factors associated with malaria, pneumonia and diarrheal diseases. Spatial patterns in the incidence of malaria, pneumonia and diarrheal diseases were mapped to identify regional heterogeneities.

**Results:**

Malaria and pneumonia were the two most common causes of mortality, with confirmed malaria and pneumonia each accounting for 20% of child deaths. Suspected and confirmed malaria accounted for 23% of child morbidity and pneumonia accounted for 17% of child morbidity. Diarrheal diseases were the cause of 7% of deaths and 10% of morbidity in children under five. Mean under-five incidence rates across all refugee camps by region were: malaria [Africa 84.7 cases/1000 U5 population/month (95% CI 67.5-102.0), Asia 2.2/1000/month (95% CI 1.4-3.0)]; pneumonia [Africa 59.2/1000/month (95% CI 49.8-68.7), Asia 254.5/1000/month (95% CI 207.1-301.8)]; and diarrheal disease [Africa 35.5/1000/month (95% CI 28.7-42.4), Asia 69.2/1000/month (95% CI 61.0-77.5)]. Measles was infrequent and accounted for a small proportion of child morbidity (503 cases, < 1%) and mortality (6 deaths, < 1%).

**Conclusions:**

As in stable settings, pneumonia and diarrhea are important causes of mortality among refugee children. Malaria remains a significant cause of child mortality in refugee camps in Africa and will need to be addressed as part of regional malaria control and elimination efforts. Little is known of neonatal morbidity and mortality in refugee settings, and neonatal deaths are likely to be under-reported. Global measles control efforts have reduced the incidence of measles among refugee children.

## Background

United Nations High Commissioner for Refugees (UNHCR) refugee camps are located predominantly in rural areas of Africa and Asia in protracted or post-emergency contexts. In these settings, refugee populations benefit from relatively stable environments and, in contrast to acute phase emergencies, mortality rates rarely exceed emergency thresholds and often are lower among refugees than surrounding host populations [1]. Factors contributing to lower mortality in refugee camps include better access to primary health care (including vaccination and nutrition programs), adequate water and sanitation, and implementation of public health measures such as disease surveillance [2-4]. The burden of disease in refugee camps is similar to that in developing countries and includes malaria, pneumonia, diarrheal disease, neonatal mortality and malnutrition [5]. Communicable diseases account for the majority of morbidity in emergency-affected populations and are often exacerbated by high rates of malnutrition [5,6]. Crowding, inadequate shelter, and poor access to water and sanitation further contribute to an increased risk of communicable diseases in camp settings. Specifically, temporary shelters and diminished vector control efforts contribute to increased malaria transmission. Pneumonia can result from overcrowding, indoor cooking fires and poor shelter construction. Improper sanitation, contamination of drinking water, and insufficient quantities of potable water increase the risk of diarrhea.

The characteristics of health facilities, nutrition programs, and access to water and sanitation are routinely monitored in UNHCR refugee camps. Much of this information is captured by the UNHCR camp registration system, ProGres, and the Health Information System (HIS). The UNHCR HIS collects standardized camp-level data and is a resource for monitoring health and disease within refugee camps in different countries in collaboration with implementing partners [7]. The HIS, established in 2006, is currently operational in refugee camps in 16 countries: Bangladesh, Burundi, Cameroon, Chad, Democratic Republic of Congo, Djibouti, Ethiopia, Kenya, Namibia, Nepal, Rwanda, Sudan, Tanzania, Thailand, Uganda and Yemen. The HIS collects data on mortality, in-patient and out-patient visits, immunization coverage, reproductive health and nutrition. Data are collected weekly and compiled into monthly reports that are entered into the HIS database. Additionally, UNHCR produces yearly factsheets for each camp, providing information on key health indicators and services that fall below standards [7,8]. Recognizing the importance of malaria, pneumonia and diarrheal diseases as major causes of child morbidity and mortality in refugee camps, we analyzed data from the UNHCR HIS to estimate the incidence and risk factors for these diseases in refugee children younger than five years of age.

## Methods

Data from 90 UNHCR refugee camps in 16 countries, including morbidity, mortality, health services and refugee health status, were obtained from the UNHCR HIS (v1.6.12.1) for the period January 2006 to February 2010 [9]. Monthly camp-level data were exported from HIS into Stata 11 (StataCorp LP, College Station, Texas, USA) and monthly averages for each camp in a given year were generated for analysis. Morbidity estimates were based on outpatient visits. Suspected and confirmed cases of malaria were combined for the morbidity analyses, and cases of watery and bloody diarrhea were combined for both morbidity and mortality analyses.

The following HIS case definitions were used: 1) suspected uncomplicated malaria was diagnosed in persons with fever or history of fever within the past 48 hours (with or without other symptoms such as nausea, vomiting and diarrhea, headache, back pain, chills or myalgia) in whom other obvious causes of fever were excluded; 2) suspected severe malaria was diagnosed in persons with symptoms as for uncomplicated malaria, as well as drowsiness with extreme weakness and associated signs and symptoms related to organ failure such as disorientation, loss of consciousness, convulsions, severe anemia, jaundice, hemoglobinuria, spontaneous bleeding, pulmonary edema and shock; 3) confirmed malaria was diagnosed in persons with uncomplicated or severe malaria with laboratory confirmation by malaria blood film or other diagnostic test for malaria parasites; 4) upper respiratory tract infection was diagnosed in persons with runny nose, cough and low grade fever; 5) pneumonia was diagnosed in children 2 months to 5 years of age with cough or difficulty breathing and breathing faster than 50 breaths/minute (2-12 months of age) or breathing faster than 40 breaths/minute (1-5 years of age); 6) watery diarrhea was diagnosed in persons with diarrhea (passage of 3 or more watery or loose stools in the past 24 hours) with or without dehydration; 7) bloody diarrhea was diagnosed in persons with diarrhea (passage of 3 or more watery or loose stools in the past 24 hours) and visible blood in the stool; 8) acute moderate malnutrition was diagnosed in children with a weight for height index of ≤ -2 and > -3 z-scores, or ≤ 80% and > 70% of median, or any child with a mid-upper arm circumference (MUAC) of > 115 mm and ≤125 mm; 9) acute severe malnutrition was diagnosed in children with a weight for height index of ≤ -3 z-scores or any child with a MUAC of ≤115 mm or any child with kwashiorkor [7].

Camp characteristics and intervention levels were examined for their association with disease incidence, and included camp size (total population and under-5 population), indicators of adequate water and sanitation (water quantity, access and proximity; latrine access and coverage; soap access), nutrition standards (global acute malnutrition and ration adequacy) and health service utilization (new visits per 10 persons/month and growth monitoring). For malaria, camp-level indictors of the prevention of malaria in pregnant women were analyzed (insecticide-treated nets [ITN] and intermittent preventive treatment for malaria in pregnancy [IPTp] coverage).

These camp characteristics were examined for outliers, zero values or inconsistencies. Outliers and inconsistent values were assumed to be reporting errors and were replaced with the average value of the two months surrounding the excluded value. Values for growth monitoring utilization, ITN ownership by pregnant women and IPTp above 100% were reset to 100%. Extreme outlier values for malaria, pneumonia and diarrhea morbidity also were replaced with the average value from the preceding and succeeding months. Camp population was modeled as a categorical variable based on terciles (0-9,999; 10,000-19,999; ≥20,000 persons). Camps were stratified into two geographic regions, Asia and Africa (including Yemen) for some analyses. Annual camp-level water and sanitation variables, including measures of access to water and latrines, and nutrition variables, including receipt of adequate food and the prevalence of undernutrition, were obtained from UNHCR annual factsheets and were converted to dichotomous variables based on performance above or below specified UNHCR standards [10].

Data analysis was conducted in Stata 11 and included summary measures of disease incidence and multivariable Poisson regression analyses to identify factors associated with disease. Camp characteristics and health status were compared between regions using t-tests for continuous variables and chi-square tests for categorical variables, using a cutoff of < 0.05 as statistically significant. Bivariate and multivariable Poisson regression models with random effects were constructed for malaria, pneumonia and diarrheal disease as dependent variables. UNHCR camp was used as the clustering variable and the offset was the average monthly camp population of children under five in each year. Standard errors in the multivariable models were calculated using bootstrapping with 1000 repetitions to correct for correlations between repeated measures in each camp.

The bivariate and multivariate analyses were restricted to those camps in 2007-2009 for which at least 8 months of data were available in a given year to account for potential seasonality in disease outcomes. Not all camps were included in the HIS in 2006 and complete HIS datasets were not available for 2010 at the time of analysis. These criteria excluded three countries from bivariate and multivariate analyses (Democratic Republic of Congo, Djibouti and Namibia) and restricted the analysis to 80 of the 90 camps. The Poisson models for malaria were further restricted to camps with an average monthly malaria incidence rate of 4 or more cases/1000 under five population/month in a given year to exclude camps in regions where malaria transmission was minimal or absent. All camps were included in the proportional morbidity and mortality assessments and GIS mapping.

ArcGIS 9.2 (Redlands, CA) was used to map the incidence of malaria, pneumonia and diarrheal disease and assess regional heterogeneities. Camp incidence rates were displayed over projections of malaria parasite prevalence from the Malaria Atlas Project (MAP) [11] or the country under-five mortality rates from the 2007 UNICEF State of the World's Children's Reports [12]. Camp-level period incidence rates were calculated using all reported cases in children younger than five years from 2006 to 2010, and are reported as cases per 1000 children younger than five years per month. The mapped incidence rates were divided into quintiles as indicated by the size and color of the circles.

## Results

### Camp characteristics

Because UNHCR had more refugee camps in Africa than Asia, 117 camp-years were analyzed for Africa and 36 for Asia. Camp characteristics in Africa and Asia were significantly different (Table 1), with larger camps more common in Africa. Health facility visits (adjusted for population size) (0.29 visits/person/months vs. 0.15, p < 0.001) and growth monitoring (68.4% vs. 37.3%, p < 0.001) were more common in camps in Asia than Africa (Table 1). Camps in Asia also met standards for water and sanitation services more frequently than those in Africa (e.g. quantity of water, person per water tap, distance to water source and percentage of families with a latrine). As an overall indicator of child health, malnutrition was more prevalent in Africa (48.6% of camps with ≥10% global malnutrition) than Asia (25.0%, p = 0.014) (Table 1).

**Table 1:** Characteristics of UNHCR refugee camps by region^a^

	Africa (n = 117)^b^	Asia (n = 36)	p-value^c^
Camp Population^d^			
Total population (%)			**0.007**
< 10,000	26.5	25.0	
10,000-< 20,000	37.6	63.9	
≥20,000	35.9	11.1	
Under five (U5) population [mean (95% CI)]	3812 (3180-4445)	1761 (1405-2117)	**< 0.001**
**Incidence Rates (U5 cases/1000 population/month)^d^**			
Malaria	84.7 (67.5-102.0)	2.2 (1.4-3.0)	**< 0.001**
Pneumonia	59.2 (49.8-68.7)	254.5 (207.1-301.8)	**< 0.001**
Diarrhea	35.5 (28.7-42.4)	69.2 (61.0-77.5)	**< 0.001**
**Health Facility Utilization^d^**			
New visits/person/month	0.15 (0.13-0.16)	0.29 (0.27-0.32)	**< 0.001**
Growth monitoring utilization (%)	37.3 (31.4-43.1)	64.8 (55.2-74.3)	**< 0.001**
**Malaria Prevention during Pregnancy^d^**			
Insecticide treated net coverage (ITN) (%)	50.4 (43.4-57.5)	10.2 (1.4-18.9)	**< 0.001**
Intermittent preventative treatment (IPTp) (%)	68.7 (62.5-74.9)	0.78 (0.03-1.5)	**< 0.001**
**Water & Sanitation (%)^e^**			
Average quantity of potable water/person/day			
≤20 L	62.4	8.3	**< 0.001**
> 20 L (standard)	37.6	91.7	
# of persons per usable water tap			
≥80	82.5	50.0	**< 0.001**
< 80 (standard)	17.5	50.0	
Living within 200 m from water point (%)			
< 100%	64.3	2.8	**< 0.001**
100% (standard)	35.7	97.2	
# of persons per communal latrine			
> 20	51.6	52.8	0.900
≤20 (standard)	48.4	47.2	
Families with latrines (%)			
< 100%	88.4	29.4	**< 0.001**
100% (standard)	11.6	70.6	
Families receiving > 250 g soap/person/mo (%)			
< 90%	36.2	41.2	0.636
≥90% (standard)	63.8	58.8	
**Nutrition (%)^e^**			
Global acute malnutrition prevalence (%)			
≥10%	48.6	25.0	**0.014**
< 10% (standard)	51.4	75.0	
Average kCals/person/day^f^			
< 2100	34.0	22.2	**0.189**
≥2100 (standard)	66.0	77.8	

^a^Categorical variables are shown as % and continuous variables are shown as mean (95% confidence intervals)

^b^Africa region includes Yemen; n = number of camp-years for the two regions; data is only included if there are at least 8 months of data per year, for the years 2007-2009

^c^p-values calculated using t-test for continuous variables and χ^2 ^for categorical variables

^d^Values directly from HIS database or calculated based on HIS values reported for outpatient visits

^e^From 2007-2009 Annual HIS Factsheets

^f^kCals/person/day was reported as average in 2008-9 and minimum in 2007.

### Causes of Child Morbidity and Mortality

**Figure 1:**
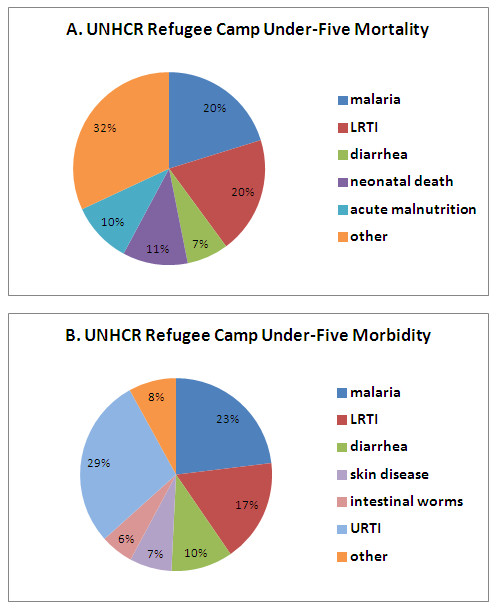
**Cause-specific morbidity and mortality in children younger than five years of age for refugee camps in the UNHCR HIS database, January 2006 to February 2010**. A. Mortality for all recorded causes. Cases of watery and bloody diarrhea were combined. B. Out-patient visits for major causes of morbidity within refugee camps. Suspected and confirmed cases of malaria were combined, as were cases of watery and bloody diarrhea. Only causes accounting for 5% or more of mortality or morbidity are shown, with the remaining causes listed as "Other".

The leading causes of morbidity and mortality in children younger than five years of age in the UNHCR refugee camps were malaria, pneumonia and diarrheal disease (Figure 1). Malaria and pneumonia were the two most common causes of mortality overall, with confirmed malaria and pneumonia each accounting for 20% of child deaths. Suspected and confirmed malaria accounted for 23% of child morbidity, whereas pneumonia accounted for 17% of child morbidity. Diarrheal diseases were the cause of 7% of deaths and 10% of morbidity in children under five.

Mean under-five incidence rates across all refugee camps by region were: malaria [Africa 84.7 cases/1000 U5 population/month (95% CI 67.5-102.0), Asia 2.2/1000/month (95% CI 1.4-3.0)]; pneumonia [Africa 59.2/1000/month (95% CI 49.8-68.7), Asia 254.5/1000/month (95% CI 207.1-301.8)]; and diarrheal disease [Africa 35.5/1000/month (95% CI 28.7-42.4), Asia 69.2/1000/month (95% CI 61.0-77.5)].

**Figure 2:**
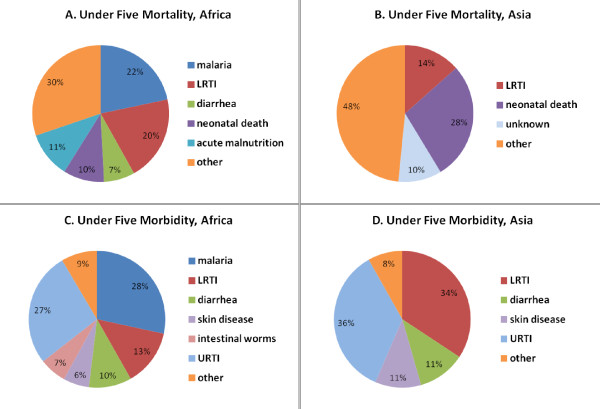
**Major causes of mortality and morbidity in children younger than five years of age by region**. Mortality is shown for all listed causes in (A) African and (B) Asian UNHCR refugee camps. Cases of watery and bloody diarrhea were combined. (C and D) Out-patient visits for the major causes of morbidity in UNHCR refugee camps by region. Suspected and confirmed malaria cases, and watery and bloody diarrhea, were combined. Only those causes accounting for 5% or more of mortality or morbidity are shown, with the remaining causes listed as "Other".

Causes of mortality varied by region. Malaria was the leading cause of death in camps in Africa but accounted for no deaths in Asia. Pneumonia was a major cause of death in both regions but had a substantially higher incidence rate in Asia than Africa. Diarrheal diseases were proportionally a more frequent cause of death in Africa than in Asia (Figure 2A and 2B). Neonatal deaths (within the first 28 days of life) were more prevalent in Asia (28% of under five deaths) and acute malnutrition as a cause of death was more common in Africa (11%) (Figure 2A and 2B).

Regional variations in morbidity also were identified (Figure 2C and 2D). In Africa, malaria was the leading cause of clinical visits, accounting for 28% of health facility consultations among children under five. Diarrheal diseases were a leading cause of under five morbidity in both regions, and pneumonia was more prevalent in Asia than Africa (Figure 2C and 2D). Although not severe, upper respiratory tract infections accounted for a significant proportion of morbidity (29%) (Figure 1). Skin diseases and intestinal worms were other causes of morbidity in children under five. Surprisingly, measles accounted for only a small proportion of child morbidity (503 cases, < 1%) and mortality (6 deaths, < 1%).

### Risk factors for child morbidity

Region and recent health facility visits were significantly associated with malaria incidence (Table 2): the incidence of malaria in Africa was 40-100 times greater than in Asia (Table 1 p < 0.001). Neither ITN ownership during pregnancy (IRR = 0.74, 95% CI 0.44-1.24) nor IPTp with two doses of sulfadoxine-pyrimethamine (IRR = 1.12, 95% CI 0.25-4.99) was significantly associated with the incidence of malaria. Health service utilization (number of new visits/10 persons) was associated with increased risk of malaria (IRR = 2.57, 95% CI 1.48-4.48), which may reflect increased care seeking for children with malaria (Table 2).

**Table 2:** Incidence rate ratios for malaria^a^

	Malaria^b^	
	Unadjusted	Adjusted^c^
**Camp Location & Size**		
Small (< 10,000)	Reference	Reference
Medium (10,000-19,999)	0.85 (0.56-1.28)	1.94 (0.69-5.46)
Large (≥20,000)	0.89 (0.55-1.45)	2.01 (0.57-7.13)
Africa^d^	Reference	Reference
Asia	**0.06 (0.04-0.10)**	**0.01 (0.001-0.13)**
**Water and Sanitation (reference category is below the specified standard)^e^**		
Water Quantity	**1.33 (1.05-1.67)**	1.11 (0.60-2.08)
Water Access	1.39 (0.82-2.35)	1.66 (0.76-3.65)
Water Proximity	2.34 (0.66-8.21)	0.99 (0.63-1.56)
Latrine Access	1.21 (0.67-2.19)	1.07 (0.74-1.53)
Latrine Coverage	1.20 (0.84-1.73)	0.87 (0.33-2.26)
Soap Access	0.87 (0.63-1.22)	N/A^f^
**Nutrition Standards (reference category is below the specified standard)^e^**		
Global Acute Malnutrition	**1.29 (1.04-1.61)**	1.73 (0.72-4.17)
Ration Adequacy	1.01 (0.80-1.28)	0.62 (0.25-1.56)
**Health Service Utilization**		
New Visits (per 10 persons/month)	**1.92 (1.23-3.01)**	**2.57 (1.48-4.48)**
Growth Monitoring (%)^g^	**0.98 (0.97-0.99)**	0.99 (0.98-1.01)
**Prevention of Malaria in Pregnancy**		
Insecticide Treated Nets (ITNs)	0.60 (0.17-2.06)	0.74 (0.44-1.24)
Intermittent Preventive Treatment (IPTp)	0.94 (0.51-1.74)	1.12 (0.25-4.99)

^a^Bold indicates statistically significant results (p < 0.05)

^b^The analysis was restricted to camps having an annual average monthly malaria incidence rate of 4 or more cases/1000 population/month in a given year and at least 8 months of data in a given year

^c^The adjusted model included all of the variables shown.

^d^Africa includes Yemen

^e^The IRR compares meeting or exceeding the standard to falling below the standard. The reference categories for UNHCR performance standards and indicators are as follows: water quantity > 20 L/person/day; water access < 80 persons per tap; water proximity 100% within 200 m; latrine access ≤20 person per latrine; latrine coverage 100% of households; soap access ≥90% of households with > 250 g soap/person/month; global acute malnutrition prevalence < 10%; and average kCals per person per day ≥2100 (in 2007 the indicator was minimum kCals per person per day).

^f^Access to soap was only reported for 2008 and 2009. So as to not exclude the 2007 data, soap was not included in the adjusted model.

^g^IRR for every 1% increase in growth monitoring utilization

The incidence of pneumonia was associated with proximity to a water source, camp population size and new visits to a health facility (Table 3). Compared to Africa, camps in Asia had a higher risk of pneumonia (IRR = 4.52, 95% CI 3.18-6.41); however, this was not significant after adjusting for other camp characteristics. Large camps with populations ≥20,000 were associated with a significantly increased risk for pneumonia compared to small (< 10,000) camps (IRR = 2.07, 95% CI 1.03-4.15). As for malaria, a higher number of new patient visits to a health facility was associated with an increased risk for pneumonia (IRR = 1.73, 95% CI 1.26-2.37), perhaps reflecting increased care seeking.

**Table 3:** Incidence rate ratios for pneumonia and diarrhea^a^

	Pneumonia		Diarrhea	
	Unadjusted	Adjusted^b^	Unadjusted	Adjusted^b^
**Camp Location & Size**				
Small (< 10,000)	Reference	Reference	Reference	Reference
Medium (10,000-19,999)	1.11 (0.89-1.39)	1.43 (0.90-2.26)	1.09 (0.85-1.39)	**1.80 (1.07-3.03)**
Large (≥20,000)	1.35 (0.88-2.08)	**2.07 (1.03-4.15)**	1.48 (0.98-2.25)	**2.16 (1.04-4.49)**
Africa^c^	Reference	Reference	Reference	Reference
Asia	**4.52 (3.18-6.41)**	1.65 (0.79-3.43)	**1.93 (1.52-2.45)**	0.77 (0.35-1.71)
**Water and Sanitation (reference category is below the specified standard)^d^**				
Water Quantity	1.04 (0.70-1.53)	1.06 (0.66-1.70)	1.01 (0.53-1.94)	0.95 (0.66-1.37)
Water Access	1.32 (0.94-1.86)	1.04 (0.77-1.40)	1.33 (0.94-1.89)	1.27 (0.85-1.89)
Water Proximity	**1.32 (1.12-1.54)**	**1.38 (1.06-1.81)**	0.84 (0.55-1.28)	1.03 (0.74-1.44)
Latrine Access	0.92 (0.82-1.03)	0.91 (0.79-1.06)	0.91 (0.74-1.13)	1.04 (0.83-1.31)
Latrine Coverage	0.77 (0.49-1.22)	0.77 (0.49-1.21)	0.81 (0.61-1.08)	0.97 (0.65-1.46)
Soap Access	0.85 (0.62-1.16)	N/A^e^	0.89 (0.72-1.10)	N/A^e^
**Nutrition Standards (reference category is below the specified standard)^d^**				
Global Acute Malnutrition	**0.86 (0.75-0.98)**	1.01 (0.74-1.37)	0.94 (0.78-1.14)	1.00 (0.68-1.46)
Ration Adequacy	1.06 (0.85-1.31)	1.18 (0.86-1.63)	**1.18 (1.01-1.37)**	1.09 (0.79-1.51)
**Health Service Utilization**				
New Visits (per 10 persons/month)	**1.71 (1.42-2.05)**	**1.73 (1.26-2.37)**	1.31 (0.99-1.74)	**1.90 (1.38-2.62)**
Growth Monitoring (%)^f^	1.00 (0.99-1.00)	1.01 (1.00-1.02)	1.01 (1.00-1.01)	1.01 (1.00-1.01)

^a ^Bold indicates statistically significant results (p < 0.05)

^b^The adjusted model included all of the variables shown, but was restricted to camps in 2007-2009 that had at least 8 months of data in a given year.

^c ^Africa includes Yemen

^d ^The IRR compares meeting or exceeding the standard to falling below the standard. The reference categories for UNHCR performance standards and indicators are as follows: water quantity > 20 L/person/day; water access < 80 persons per tap; water proximity 100% within 200 m; latrine access ≤20 person per latrine; latrine coverage 100% of households; soap access ≥90% of households with > 250 g soap/person/month; global acute malnutrition prevalence < 10%; and average kCals per person per day ≥2100 (in 2007 the indicator was minimum kCals per person per day).

^e^Access to soap was only reported for 2008 and 2009. So as to not exclude 2007 data, soap was not included in the adjusted model.

^f ^IRR for every 1% increase in growth monitoring utilization

Camp size and new patient visits were also significantly associated with diarrheal disease in multivariable analyses (Table 3). Camps in Asia were more likely to have cases of diarrheal disease than those in Africa (IRR = 1.93, 95% CI 1.52-2.45); however, region alone was not significant after accounting for other camp characteristics. Camps with large (≥20,000 refuges) and medium (10,000-19,999 refugees) size populations were associated with increased patient visits for diarrhea (IRR = 2.16, 95% CI 1.04-4.49 and IRR = 1.80, 95% CI 1.07-3.03, respectively) compared to small (< 10,000 persons) camps. Again, increased new patient visits was associated with an increase in patient visits for diarrhea (IRR = 1.90, 95% CI 1.38-2.62). In the unadjusted and adjusted models, none of the indicators of water and sanitation were significantly associated with diarrheal disease (Table 3).

### Mapping disease incidence rates

**Figure 3:**
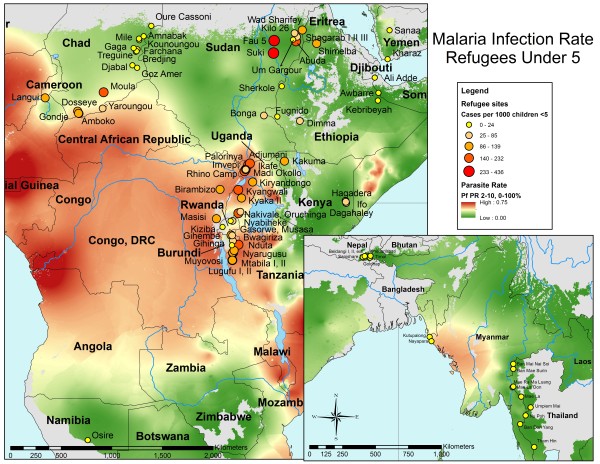
**Incidence of malaria in UNHCR refugee camps in Africa and Asia**. The incidence rate for malaria (cases per 1000 under five population per month) during the period January 2006 to February 2010 in refugees under five years old in the UNHCR camps is shown by circles. The background represents the parasite prevalence in children 2 to 10 years of age from the Malaria Atlas Project. The size and color of the circles reflect the incidence rate quintiles for the refugee camps.

**Figure 4:**
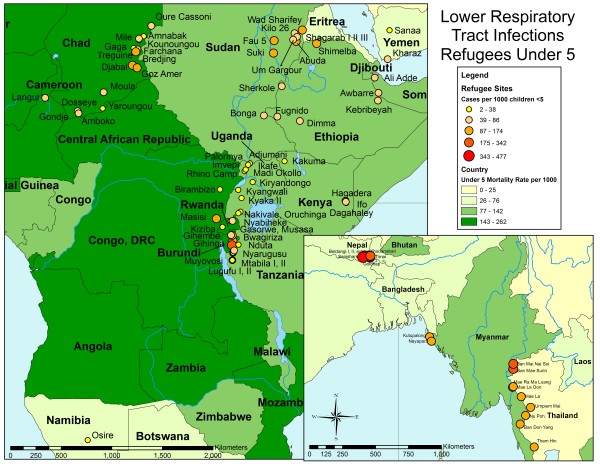
**Incidence of pneumonia in UNHCR refugee camps in Africa and Asia**. The incidence rate for pneumonia (cases per 1000 under five population per month) during the period January 2006 to February 2010 in refugees under five years old in the UNHCR camps is shown over the national under five mortality rate for each country obtained from the 2007 UNICEF State of the World's Children Report. The size and color of the camps reflects the incidence rates divided into quintiles. The country mortality rate is shown as shades of green.

**Figure 5:**
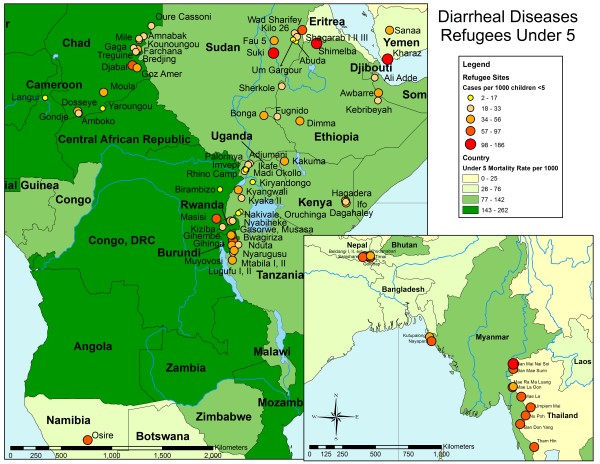
**Incidence of diarrhea in UNHCR refugee camps in Africa and Asia**. The incidence rate for diarrheal diseases (cases per 1000 under five population per month) during the period January 2006 to February 2010 in refugees under five years old in the UNHCR camps is shown over the national under five mortality rate for each country obtained from the 2007 UNICEF State of the World's Children Report. The size and color of the camps reflects the incidence rates divided into quintiles. The country mortality rate is shown as shades of green.

To assess spatial heterogeneities across UNHCR camps, disease incidence was mapped. Average malaria incidence (new cases per 1000 under-five population per month) for the period January 2006 to February 2010 in UNHCR camps was overlaid on the parasitemia rate of *Plasmodium falciparum *in children between 2 to 10 years-old obtained from the Malaria Atlas Project (Figure 3). With the exception of several camps in Sudan, camps located in areas of Asia and northeastern Africa with low PfPR_2-10 _also had low rates of malaria in UNHCR camps. For example, in Chad the incidence rates of malaria were consistent with the underlying parasite prevalence in the area. The average incidence rates of pneumonia and diarrheal disease (new cases per 1000 under-five population per month) in UNHCR camps for the period January 2006 to February 2010 were overlaid on host-country, under-five mortality rates obtained from the UNICEF State of the World's Children Report in 2007 (Figures 4 and 5). These maps highlight the heterogeneity at both the country and regional level in pneumonia and diarrheal disease incidence rates at UNHCR camps and their relationship to under-five mortality rates at the national level.

## Discussion

Analysis of UNHCR HIS data from 90 refugee camps in 16 countries indicated that the primary causes of mortality among camp-based refugee children younger than five years of age were malaria (20%), pneumonia (20%), diarrheal disease (7%), neonatal deaths (11%) and acute malnutrition (10%). The finding that the causes of under-five mortality in refugee and emergency settings are comparable to countries with high levels of child mortality has been observed previously [1,13,14]. However, the proportions of deaths due to diarrhea and neonatal causes at UNHCR camps were lower than expected and that due to malaria higher than global estimates (the latter reflecting, in part, the preponderance of UNHCR camps in Africa). Cause-specific mortality among children under-five worldwide include pneumonia (18%), diarrhea (15%), malaria (8%), and neonatal causes (41%) [15], with undernutrition contributing to half of under-five deaths.

There are several potential explanations for these differences. Little is known of neonatal morbidity, mortality and care practices in refugee settings. Neonatal deaths are likely to be underreported at these camps, contributing to the relatively low proportion of deaths due to neonatal causes. Efforts to improve access to maternal and newborn care services also could have contributed. The relatively low proportion of deaths due to diarrhea may reflect improved access to water and sanitation in these refugee settings, although no associations were observed. In contrast, despite reductions in the burden of malaria in parts of sub-Saharan Africa [16], malaria remains an important cause of child mortality among refugees, in part reflecting the location of refugee camps. Data on the proportion of children younger than five years of age sleeping under an ITN or the use of artemisinin-combination therapy to treat malaria were not available.

Historically, measles was a major cause of child mortality in emergencies and refugee camps [6,14,17] but reductions in global measles incidence and mortality likely decreased the risk of measles in refugee populations [18,19]. In addition, measles vaccine coverage is likely higher in these post-emergency camps. In contrast to many acute emergencies [20], violence and trauma were not major causes of child morbidity or mortality.

Nutritional status and indicators of water and sanitation were hypothesized to be associated with disease incidence, particularly for diarrheal diseases, and indicators of access to water, sanitation and food, as well as levels of acute malnutrition, were assessed as predictors of disease incidence using UNHCR performance standards. However, no measures of water and sanitation were significantly associated with disease incidence (with the exception of proximity to a water source and the risk of pneumonia, which is likely not a causal association). Water quantity and accessibility were assessed, but water quality was not addressed. A previous study of refugee camps in sub-Saharan Africa highlighted the burden of diarrhea morbidity and mortality attributable to incomplete water and sanitation and the fact that mortality due to diarrhea was lower in camps than in the host country [21]. As shown in the maps, striking differences in disease incidence were observed at some UNHCR camps located in close proximity, and contrasted with national background rates of disease, suggesting that multiple local factors contribute to disease incidence within a given camp.

Few large-scale studies of refugee children exist for comparison. One prior study of 51 post-emergency phase refugee camps in seven countries assessed predictors of crude and under-five mortality [3]. Contrary to the association of larger camp size with pneumonia and diarrhea in these UNHCR camps, larger camp size was associated with a lower risk of adverse health outcomes in the earlier study. Risk factors for higher crude mortality included newly opened camps, proximity to conflict regions and increased travel time to referral hospitals. Increased under-five mortality was associated with poorer access to potable water and elevated rates of diarrheal disease. Access to sanitation (number of people per functioning latrine) was associated with reductions in crude mortality but no statistically significant association was observed between access to latrines and under-five mortality, similar to the findings in these UHNCR camps.

The HIS was designed to provide basic information on refugee health status and services provided by health facilities in camps. Missing data, particularly for seasonal infectious diseases, may have biased morbidity and mortality estimates. Because the covariates were camp-level characteristics, associations between risk factors for disease and disease incidence may not have been observed. Individual-level data may be necessary to detect these associations.

## Conclusions

As in stable settings, pneumonia and diarrhea are important causes of mortality among refugee children. Malaria remains a significant cause of child mortality in refugee camps in Africa and will need to be addressed as part of regional malaria control and elimination. Little is known of neonatal morbidity and mortality in refugee settings, and neonatal deaths are likely to be under-reported. Global measles control efforts have reduced the incidence of measles among refugee children.

## List of Abbreviations

HIS: health information system; IRR: incidence rate ratio; ITN: insecticide-treated net; IPTp: intermittent preventive treatment in pregnancy; MAP: Malaria Atlas Project; MUAC: mid-upper arm circumference; UNHCR: United Nations High Commissioner for Refugees

## Competing interests

The authors declare that they have no competing interests.

## Authors' contributions

CLH conducted the analyses and drafted the manuscript. SD conceived of the study and participated in the design, coordination and drafting of the manuscript. JA assisted with the spatial mapping. CH conceived of the study and participated in the design, coordination and drafting of the manuscript. PS conceived of the study and participated in the design, coordination and drafting of the manuscript. WJM participated in the design, coordination and drafting of the manuscript. All authors have read and approved the final manuscript.
